# Vibrational entropy of disorder in Cu_3_Au with different degrees of short-range order

**DOI:** 10.1039/c8cp01656a

**Published:** 2018-07-11

**Authors:** A. Benisek, E. Dachs, M. Grodzicki

**Affiliations:** a Chemistry and Physics of Materials , University of Salzburg , Austria . Email: artur.benisek@sbg.ac.at

## Abstract

L1_2_ ordered Cu_3_Au and fcc-disordered samples with different degrees of short-range order were synthesised by annealing and/or quenching experiments. Low-temperature heat capacities were determined by relaxation calorimetry. From these data the vibrational entropy of disorder was derived. The calorimetric results show that the vibrational entropy does not depend on the degree of short-range order. The calorimetric investigations were complemented by density functional calculations with different functionals simulating various atomic configurations by super cells of different size. Using super cells containing 32 atoms, the computed entropies show only small variations with the change of short-range order in good agreement with the calorimetric results. Using, however, super cells with only 8 atoms, the results depend strongly on the chosen atomic configuration at variance with the calorimetric data. This result is important for investigating substances with larger molecules (*e.g.*, silicate solid solutions) because such investigations are typically limited on super cells containing only a few sites on which substitution takes place.

## Abstract 2

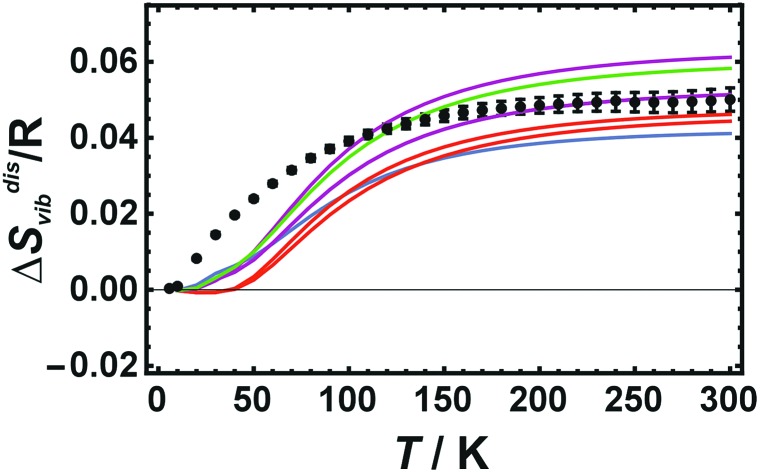
The vibrational entropies of disorder in different short-range ordered Cu_3_Au were studied by calorimetric and DFT methods.

## Introduction

The atomic distribution of Au and Cu in Cu_3_Au changes as a function of temperature. At low temperatures a L1_2_ ordered phase (with *Pm*3[combining macron]*m* symmetry) is stable with Au occupying the corner and Cu the face centres of the cubic unit cell.^1^ At about 660 K, Cu_3_Au undergoes a diffusive phase transition, where some Au and Cu atoms change their positions by chemical diffusion leading to the face-centred cubic (fcc) structure with *Fm*3[combining macron]*m* symmetry. The atomic distribution is then disordered, although there is some short-range order (SRO) decreasing with further increasing the temperature. Ordered and disordered samples can be synthesised by annealing and quenching experiments. Their atomic distributions are in equilibrium with the annealing temperature from which they were quenched.

The fcc-structure contains a single crystallographic site, *i.e.*, all crystallographic sites in the unit cell are equivalent. The situation with an fcc-alloy is more complicated. Although the crystallographic sites are different, in general, thermodynamic averaging, however, yields a single averaged crystallographic site. Accordingly, a disordered Cu_3_Au crystal exhibits thus only acoustic modes and the vibrational characteristics are completely different compared to an ordered Cu_3_Au crystal where two different crystallographic sites generate both acoustic and optical modes.^2^ Optical modes require a long-range pattern. Such modes generated in a given disordered unit cell will be extinguished in the neighboured cells because of their different atomic configurations. In first-principles studies, the disordered state is often modelled by a so-called special quasirandom structure (SQS). It has been reported that unit cells with only 8 atoms provide a good approximation to the disordered state.^3^ The vibrational behaviour of fcc alloys approximated by such unit cells is characterised by optical modes because the calculations are based on the assumption that the whole crystal is built up by cells with this particular atomic configuration. The question is raised, if 8 atoms are actually sufficient to obtain acceptable results for different disordered states.

The differences in enthalpy and vibrational entropy between fully ordered and fully disordered Cu_3_Au have already been investigated by calorimetric studies^4^–^7^ as well as by *ab initio* methods^8^–^13^ and are summarised in Table 1.

**Table 1:** Difference in enthalpy (Δ*H*^dis^) and vibrational entropy (Δ*S*disvib) between fully ordered and fully disordered Cu_0.75_Au_0.25_ from the average of the following calorimetric (Ref.^cal^) and *ab initio* studies (Ref.^ab^)

	Mean values ± standard dev.	Ref.^cal^	Ref.^ab^
Δ*H*^dis^/kJ mol^–1^	3.9 ± 0.3	5 and 6	8 and 13
Δ*S*disvib/R	0.05 ± 0.03	4 and 6	9–11

The heat capacity is best suited for exploring disordering since it enables the separation of the different contributions. The difference in heat capacity between the ordered and the disordered structure below room temperature is of vibrational origin and yields the vibrational enthalpy and entropy of disorder approaching the high temperature limit asymptotically at about 300 K. Above 400 K, heating during the heat capacity measurements changes the atomic configuration. Consequently the measured heat capacity of disorder is primarily of configurational origin. Since the vibrational behaviour is modified by changes of the atomic configuration, the heat capacity of disorder above 400 K produces a change in enthalpy and entropy, which contains both vibrational and configurational contributions.^6^

Studies investigating the influence of SRO on these properties of Cu_3_Au are rare. Whereas the impact on the enthalpy is known and amounts to about 1.5 kJ mol^–1^ when heating the sample from the phase transition at 680 K to 1000 K, the influence on the vibrational entropy investigated in our former study^6^ was not conclusive. Our results could be interpreted both as a slight increase or decrease of the vibrational entropy with increasing disorder since the uncertainties in the measured vibrational entropy did not allow a clear distinction between these two possibilities. A recent theoretical study^14^ on Cu_3_Au using a pseudo-atomic approach, however, proposed large differences in the vibrational entropy with regard to different degrees of SRO. The values for the vibrational entropy of disorder ranged from 0.03 to 0.16 R with increasing disorder. Another *ab initio* study^15^ of the vibrational entropy as a function of the degree of SRO in a Fe–Cr alloy shows also a systematic increase of the vibrational entropy with increasing disorder. However, instead of using a super cell for simulating the random alloy dynamical matrix, the authors used different bond lengths with different ordered structures.^15^

In this study, additional low-temperature heat capacity measurements were carried out using an improved experimental setup. The experimental uncertainties were significantly reduced allowing for the first time to resolve conclusively the dependency between the vibrational entropy of disorder and the degree of SRO. Additionally, density functional calculations using ordered *Pm*3[combining macron]*m* and various disordered Cu_3_Au super cells were performed and the results were compared to the calorimetric ones. This comparison was used to address the question of the required size of the super cell.

## Experimental

### Cu_3_Au samples

Copper and gold powders (purity of >99.9%) were mixed in an agate mortar, pressed to a pellet and melted at 1373 K in an evacuated quartz-glass ampoule, which was used in all subsequent heating experiments. The melted sample was quenched and then pressed to a flat disc and again held at 973 K for 2 days. To produce samples with defined atomic distributions, the sample was equilibrated at different temperatures and quenched into an iced brine bath. The heat capacity measurements started immediately after quenching. The most ordered sample was heated to 783 K then cooled to 658 K where it was held for 1 day followed by further cooling steps: *T* = 628 K for 2 days, *T* = 598 K for 4 days, and *T* = 568 K for 4 days. Finally, the furnace was turned off and slowly cooled down to room temperature. The sample prepared in this way is characterised by strong X-ray superlattice diffraction peaks indicating an ordered Cu–Au distribution. The X-ray patterns of the disordered and ordered samples are shown elsewhere.^6^ The corresponding lattice parameters are *a*_0_/nm = 0.37561 ± 0.00001 and 0.37456 ± 0.00001, respectively. Both values are slightly larger than those of Okamoto *et al.*,^1^ who found *a*_0_/nm = 0.375324 and 0.37426 for disordered and ordered samples, respectively. Using their relationship between composition and lattice parameter for disordered Cu_3_Au, a copper mole fraction of *X*_Cu_ = 0.745 is calculated from the observed *a*_0_ value. This composition was confirmed by an electron microscopic investigation exhibiting the sample as homogeneous and stoichiometric within experimental uncertainties.

### Relaxation calorimetry (PPMS)

Low-temperature heat capacities from 5 to 300 K were measured using a commercially available relaxation calorimeter (heat capacity option of the PPMS by Quantum Design®). A piece with *ca.* 3.3 × 3.3 × 0.25 mm (∼30 mg) was polished on one side and mounted onto the calorimeter platform using Apiezon N grease. This surface was reprocessed until a tight thermal contact between sample and calorimeter platform was achieved. Particular attention was payed to the flatness of this surface. In all further measurements, the sample was mounted onto the platform with the same surface in contact with the calorimeter platform. The thermal contact between sample and calorimeter platform denoted as sample-coupling routinely fitted in the evaluation procedure of the raw PPMS data was similar from run to run. Accordingly, the uncertainties resulting from possibly different thermal contacts when comparing different runs^16^,^17^ were minimised.

### Differential scanning calorimetry (DSC)

The state of the order/disorder of the sample was checked after the PPMS runs by a DSC method measuring the heat capacity between 300 and 720 K. The data were used to calculate the enthalpy change due to ordering/disordering during the DSC run and were compared to those derived earlier.^6^

### Evaluation of the raw heat capacity data

In order to calculate the vibrational entropy, the measured low temperature heat capacities were integrated numerically using a spline interpolation function of Mathematica®. The relative uncertainty of the entropy derived from the PPMS heat capacity data are usually 0.2% for single-crystal and sintered powder samples as determined by a Monte Carlo technique in a previous study.^18^ In this study, the uncertainty due to the sample coupling was eliminated by the following procedure. The reprocessing of the sample surface (see above) with subsequent heat capacity measurements yielded data with slightly different values of entropy and sample-coupling displaying a linear relationship between them. It was, therefore, easy to normalise the entropy values to a single sample-coupling value. The correction factors ranged between 0.9994 and 1.0005. The remaining uncertainty in the entropy was evaluated using the uncertainties in the heat capacity and temperature as determined routinely by the PPMS software. A Monte Carlo technique was then used for the error propagation resulting in the final uncertainty of the entropy determined as low as 0.02%. This small value was confirmed by repeating the whole experiment at 703 K, *i.e.*, three quenching experiments, from which the heat capacities were measured three times at 60 temperature steps.

### Calculations by density functional theory (DFT)

Quantum-mechanical calculations were based on the DFT plane wave pseudopotential approach implemented in the CASTEP code^19^ included in the Materials Studio software from Accelrys®. The calculations were performed using the local density approximation (LDA)^20^ for the exchange–correlation functional. In addition to the LDA calculations, a gradient-corrected functional (GGA-PBE)^21^ and its revised form for solids (GGA-PBESOL)^22^ were used for comparison reasons. The valence shell consists of 3d^10^4s^1^ and 5d^10^6s^1^ electrons for Cu and Au, respectively. Structural relaxation was accounted for using a threshold for the force on each atom of 0.01 eV Å^–1^. The spacing for the *k*-point sampling was 0.02 for energy calculations and 0.03 Å^–1^ for phonon calculations. Convergence was tested by using a denser *k*-point grid. The lattice dynamics calculations were based on the finite displacement approach implemented in CASTEP calculating the forces on perturbed configurations in a super cell with positive and negative displacements. The volume of this extended super cell was 8 times larger than the super cell described in the next paragraph.

### Structural models

The investigated cells contained either 32 atoms or 8 atoms. They were constructed by doubling the lattice constants of the conventional and primitive fcc cells, respectively. The calculations started with the larger cell (32 atoms) and the fully ordered L1_2_ structure (*Pm*3[combining macron]*m* symmetry) followed by calculations on a super cell (32 atoms), where 2 Cu and 2 Au atoms changed their positions relative to the original cell. This new distribution is denoted as Dis2 with two slightly different configurations Dis2a and Dis2b. Another cell containing 32 atoms was generated by replacing 3 Cu and 3 Au atoms relative to the original cell denoted as Dis3 and so on. The simulation of the fully disordered structure used a special quasi random structure^23^ (SQS). Investigations on the smaller cells (8 atoms) involved the ordered structure with *Pm*3[combining macron]*m* symmetry and four different disordered configurations.

## Results

### PPMS determined vibrational entropy at 298.15 K of different ordered/disordered Cu_3_Au samples

The vibrational entropy (*S*^vib^) at 298.15 K of Cu_0.75_Au_0.25_ as determined by the PPMS method amounts to *S*^vib^/R = 4.416 ± 0.001 for the most ordered sample. A disordered sample with a Cu–Au distribution equilibrated at 800 K and subsequently quenched to room temperature, resulted in a *S*^vib^/R value of 4.465 ± 0.001. Both values are significantly different yielding vibrational entropy of disorder, Δ*S*disvib, of 0.049 ± 0.0014 R.

**Fig. 1:**
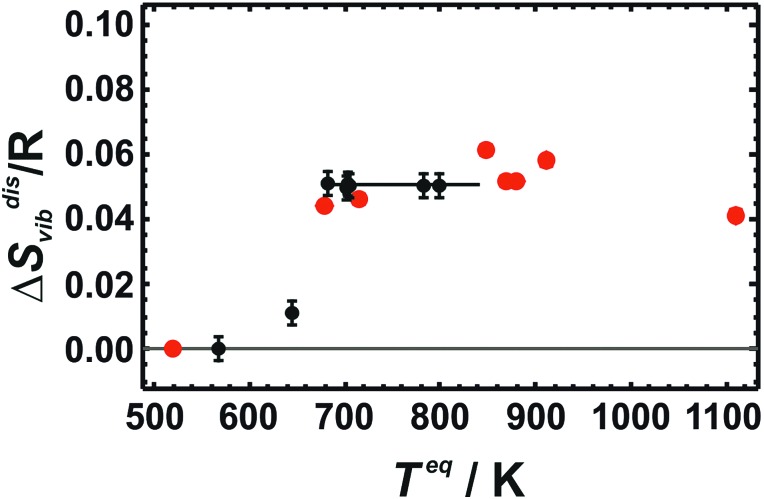
Vibrational entropies of disorder (Δ*S*disvib) of Cu_0.75_Au_0.25_ at 298.15 K. It is plotted against equilibrium temperature (*T*^eq^) as a measure of SRO. LDA calculated values using 32 atoms (red data points) are compared to the calorimetric data (black data points with error bars representing 2 sd).

Δ*S*disvib values were calculated from the calorimetric data and are plotted in Fig. 1 as a function of the equilibration temperature, *T*^eq^, defined as the temperature where the atomic distribution was equilibrated and frozen in. *T*^eq^ ranged from 568 to 800 K. The sample with an Au–Cu distribution at 568 K was the most ordered one characterised by strong X-ray super-lattice diffraction peaks.^6^ The sample at the next higher temperature (645 K) was characterised by a partially ordered Au–Cu distribution. Above 680 K, the atomic distributions of the samples were disordered (fcc-structure) with varying SRO. The sample with the highest equilibration temperature was 800 K. At even higher temperatures, the quenching process was obviously inefficient yielding samples with Au–Cu distributions that were less disordered than the one at 800 K.^6^

The results show that Δ*S*disvib does not change in the fcc-structure in the temperature range from 680 to 800 K, despite substantial increasing disorder. The increased disorder can be assessed from the change in the enthalpy of disorder increasing by 600 J mol^–1^, when the temperature is increased from 680 to 800 K.^6^ The experimentally obtained independence of *S*^vib^ on the degree of SRO may be explained by the characteristics of the fcc alloy whose vibrational behaviour is based on the presence of a single thermodynamically averaged crystallographic site. These results are at variance with a recent study^14^ on Cu_3_Au, which proposed large differences in the vibrational entropy with regard to different degrees of SRO (Δ*S*disvib ranged from 0.03–0.16 R).

### Results from LDA calculations on cells with 32 atoms

**Fig. 2:**
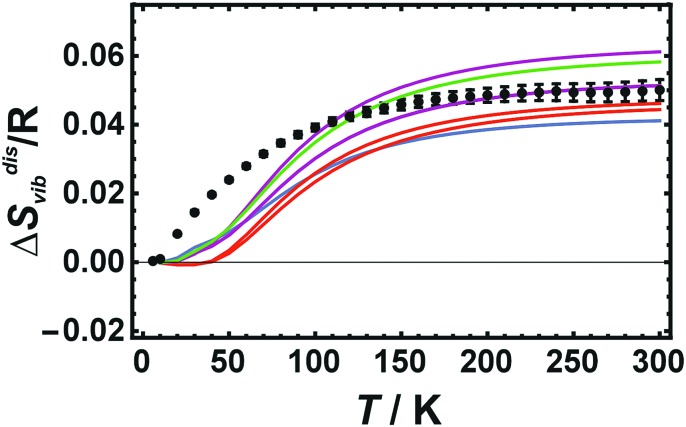
Vibrational entropies of disorder (Δ*S*disvib) of Cu_0.75_Au_0.25_ plotted against temperature. Calorimetric data with error bars (2 sd) are compared to LDA calculated data using 32 atoms (Red: Dis2a, Dis2b; Magenta: Dis3a, Dis3b, Dis3c; Green: Dis4; Blue: SQS).

Calculated Δ*S*disvib values increase with temperature in the low temperature regime (below room temperature) and reaches the high temperature limit asymptotically at room temperature as shown in Fig. 2, where LDA calculated Δ*S*disvib values using cells with 32 atoms are plotted against temperature and compared to the calorimetric ones.

**Fig. 3:**
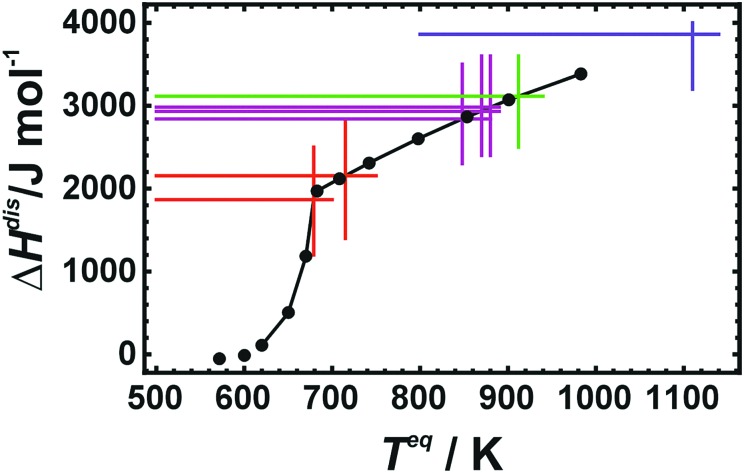
Enthalpies of disorder (Δ*H*^dis^) of Cu_0.75_Au_0.25_ plotted against equilibrium temperature (*T*^eq^). Calorimetric data^6^ are represented by black dots and lines. They are compared to LDA calculated values (Red: Dis2a, Dis2b; Magenta: Dis3a, Dis3b, Dis3c; Green: Dis4; Blue: SQS).

At low temperatures, the calculated Δ*S*disvib values are lower than the measured data but are in almost quantitative agreement at room temperature for all of the different structural models. In order to estimate the equilibrium temperature of the generated cells (Dis2, Dis3, Dis4, and SQS), the calculated energy of disorder was compared with the calorimetric enthalpy of disorder.^6^ The difference between internal energy and enthalpy of disorder, Δ*H*^dis^, at 0 K was assumed to be negligible as well as the respective heat capacities of disorder. Since these quantities are reaction energies and reaction heat capacities of solids, the above assumptions are not expected to result in noticeable errors. The results are shown in Fig. 3. The LDA energies are perfectly consistent with the calorimetric trend, *i.e.*, Δ*H*^dis^ of Dis2 is located at or just above the phase transition. Further increase in disorder (Dis3, Dis4) produced an increase in Δ*H*^dis^ and consequently of the equilibrium temperature, *T*^eq^. The SQS cell yielded an estimated *T*^eq^ of about 1100 K. Using these data, calculated Δ*S*disvib are plotted as a function of temperature and are compared to calorimetric ones in Fig. 1.

LDA calculated Δ*S*disvib agree well with the calorimetrically determined value of Δ*S*disvib/R = 0.05 with some scatter. The SQS cell yields a slightly lower Δ*S*disvib/R value of 0.042.

### Results from LDA calculations for cells with 8 atoms

**Fig. 4:**
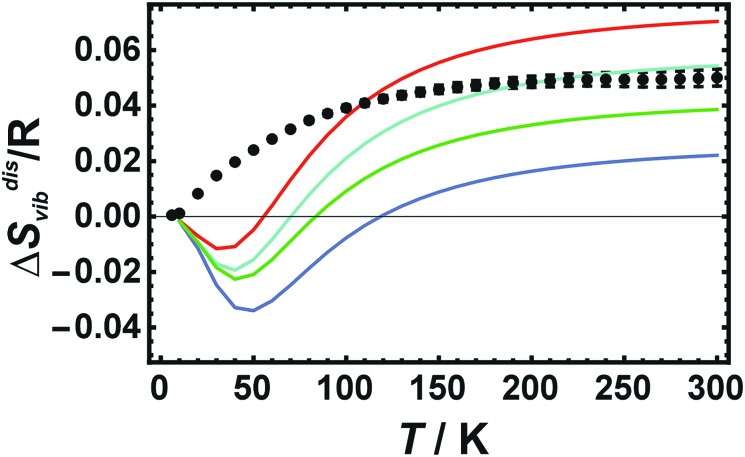
Vibrational entropy of disorder (Δ*S*disvib) of Cu_0.75_Au_0.25_ plotted against temperature. Calorimetric data with error bars (2 sd) are compared to LDA calculated data using only 8 atoms (coloured lines represent different atomic configurations).

The enthalpy of disorder for cells with 8 atoms is large showing only small differences when comparing different disordered configurations (Δ*H*^dis^ ranges from 5.78 to 5.79 kJ mol^–1^). On the other hand, the corresponding vibrational entropies exhibit strong variations when comparing different disordered configurations (Fig. 4) at variance with the calorimetric data. The agreement with the calorimetric results is particularly poor at low temperatures.

### Results using the GGA-PBE and GGA-PBESOL functionals

**Fig. 5:**
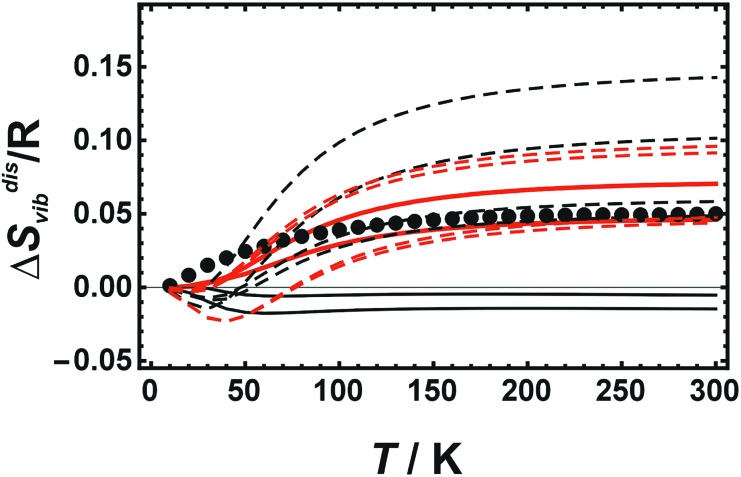
Vibrational entropy of disorder (Δ*S*disvib) of Cu_0.75_Au_0.25_ plotted against temperature. Calorimetric data (solid circles) are compared to DFT calculated data using the GGA-PBE (black lines) and the GGA-PBESOL functionals (red lines). Solid lines represent results from cells with 32 atoms (SQS and Dis2a), broken lines from cells with 8 atoms (4 different disordered configurations).

The DFT calculations were extended by applying the GGA-PBE and the GGA-PBESOL functionals to cells with 32 atoms (in addition to the ordered *Pm*3[combining macron]*m*, the fully disordered SQS structure and Dis2a as the one with the largest SRO were used), as well as to those with 8 atoms (the ordered structure and 4 disordered configurations) supplying results shown in Fig. 5.

The agreement of the GGA-PBE results with the calorimetric data is poor. The cells with 32 atoms resulted in low Δ*S*disvib values for both structures (SQS and Dis2a). The cells with 8 atoms delivered a strong variation of Δ*S*disvib with different configurations. On the other hand the GGA-PBESOL results show similar characteristics as the LDA values, *i.e.*, cells with 32 atoms gave Δ*S*disvib values agreeing for both structures (SQS and Dis2a) with the calorimetric data, whereas cells with only 8 atoms yielded again a strong variation with different configurations (Fig. 5).

## Conclusion

Unlike both the enthalpy and the configurational entropy, the vibrational entropy of Cu_3_Au does not change with varying SRO as determined by low temperature calorimetry. Using the LDA and the GGA-PBESOL functionals good agreement of calculated vibrational entropies with the calorimetric results is obtained, if super cells with 32 atoms are used comprising a total number of 256 atoms within the finite displacement approach. This result indicates that super cells with this size are large enough to simulate the presence of a single thermodynamically averaged crystallographic site and thus the characteristics of the fcc alloy. Using cells with just 8 atoms, however, the calculated entropies depend strongly on the assumed configurations. The cell is obviously too small to simulate successfully the random character of the fcc alloy. This result becomes relevant when investigating more complex systems as, *e.g.*, silicate solid solutions. Here, only a restricted number of sites where substitution takes place can be considered in the DFT calculation due to limited computing power. In our first-principles study on the vibrational entropies of the NaAlSi_3_O_8_–KAlSi_3_O_8_ solid solution, we found that the results depended strongly on the assumed atomic configurations, *viz.* disordered, ordered or clustered structures.^24^ This, however, may perhaps be assigned to the cells containing a small number of sites (4 and 16 sites) where substitution took place.

## Conflicts of interest

There are no conflicts to declare.
